# Tumor-Associated Macrophages in Glioblastoma Multiforme—A Suitable Target for Somatostatin Receptor-Based Imaging and Therapy?

**DOI:** 10.1371/journal.pone.0122269

**Published:** 2015-03-25

**Authors:** Constantin Lapa, Thomas Linsenmann, Katharina Lückerath, Samuel Samnick, Ken Herrmann, Carolin Stoffer, Ralf-Ingo Ernestus, Andreas K. Buck, Mario Löhr, Camelia-Maria Monoranu

**Affiliations:** 1 Department of Nuclear Medicine, University Hospital Würzburg, Würzburg, Germany; 2 Department of Neurosurgery, University Hospital Würzburg, Würzburg, Germany; 3 Department of Neuropathology, Institute of Pathology, University of Würzburg, Würzburg, Germany; University of Portsmouth, School of Pharmacy & Biomedical Sciences, UNITED KINGDOM

## Abstract

**Background:**

Glioblastoma multiforme (GBM) is the most common primary brain tumor in adults. Tumor-associated macrophages (TAM) have been shown to promote malignant growth and to correlate with poor prognosis. [1,4,7,10-tetraazacyclododecane-NN′,N″,N′″-tetraacetic acid]-d-Phe1,Tyr3-octreotate (DOTATATE) labeled with Gallium-68 selectively binds to somatostatin receptor 2A (SSTR2A) which is specifically expressed and up-regulated in activated macrophages. On the other hand, the role of SSTR2A expression on the cell surface of glioma cells has not been fully elucidated yet. The aim of this study was to non-invasively assess SSTR2A expression of both glioma cells as well as macrophages in GBM.

**Methods:**

15 samples of patient-derived GBM were stained immunohistochemically for macrophage infiltration (CD68), proliferative activity (Ki67) as well as expression of SSTR2A. Anti-CD45 staining was performed to distinguish between resident microglia and tumor-infiltrating macrophages. In a subcohort, positron emission tomography (PET) imaging using ^68^Ga-DOTATATE was performed and the semiquantitatively evaluated tracer uptake was compared to the results of immunohistochemistry.

**Results:**

The amount of microglia/macrophages ranged from <10% to >50% in the tumor samples with the vast majority being resident microglial cells. A strong SSTR2A immunostaining was observed in endothelial cells of proliferating vessels, in neurons and neuropile. Only faint immunostaining was identified on isolated microglial and tumor cells. Somatostatin receptor imaging revealed areas of increased tracer accumulation in every patient. However, retention of the tracer did not correlate with immunohistochemical staining patterns.

**Conclusion:**

SSTR2A seems not to be overexpressed in GBM samples tested, neither on the cell surface of resident microglia or infiltrating macrophages, nor on the surface of tumor cells. These data suggest that somatostatin receptor directed imaging and treatment strategies are less promising in GBM.

## Introduction

Glioblastoma multiforme (GBM) is the most common primary brain tumor in adults and represents approximately 65% of all newly diagnosed malignant gliomas [1]. GBM patients have an extremely poor outcome with only 10% surviving 5 years after the initial diagnosis, and a median survival of about 15 months, even after aggressive therapies including surgery, external beam radiotherapy and chemotherapy [2–4]. The limited response to standard therapies is believed to result from tumor heterogeneity as well as diffuse infiltration into the normal brain parenchyma [5].

GBM generally contains multiple morphologically diverse cell types that express neural, glial and myeloid markers [6]. In fact, mesenchymal cells with characteristics of tumor-associated macrophages (TAM) and/or microglia can comprise up to 40% of GBM [7, 8]. TAM have been shown to promote malignant glioma growth by creating a local immunosuppressive microenvironment, secreting pro-angiogenic factors and enhancing invasion mediated by the production of soluble factors such as transforming growth factor-β (TGF-β), interleukin (IL)-10, vascular endothelial growth factor, and matrix metallopeptidase-9 [9–14]. The extent of TAM infiltration has been associated with poor prognosis in patients with breast, prostate, bladder or cervical cancers [15–18]. In glioblastoma, the number of macrophages is higher compared to that observed in low-grade gliomas, and correlates with the vascular density within the tumor [19].

Somatostatin receptors (SSTR) are a family of G protein-coupled seven-transmembrane receptors of which at least five different subtypes (SSTR1-5) have been described. SSTR mediate the effects of somatostatin, a regulatory peptide produced by neuroendocrine, inflammatory, and immune cells [20]. The receptors differ in their molecular structure, tissue distribution, intracellular signaling, and pharmacological characteristics [20, 21]. [1,4,7,10-tetraazacyclododecane-NN′,N″,N′″-tetraacetic acid]-d-Phe1,Tyr3-octreotate (DOTATATE) labeled with the generator-derived positron-emitting isotope Gallium-68 (^68^Ga) selectively binds to somatostatin receptor 2A (SSTR2A) [22, 23]. ^68^Ga-DOTATATE positron emission tomography (PET) is routinely used for the staging and restaging of neuroendocrine tumors [24]. Importantly, SSTR2 was also found to be specifically expressed and up-regulated in human macrophages [25–27]. Therefore, this tracer might be suitable for the assessment of macrophage infiltration in GBM and thereby allowing risk stratification, indicating patients with a high amount of infiltrating macrophages and, as a consequence, a worse prognosis.

On the other hand, several studies have reported conflicting results concerning the expression of SSTR2A on the cell surface of malignant gliomas. Some groups found a rather robust receptor expression in human gliomas including GBM [28–30], others did not [31–32]. However, on the basis of SSTR-PET, successful treatment of GBM and meningioma using the somatostatin receptor-based radiopharmaceuticals ^90^Y-[1,4,7,10-tetraazacyclododecane-*N*,*N′*,*N″*,*N″′*-tetraacetic acid^0^-d-Phe^1^,Tyr^3^]octreotide (^90^Y-DOTATOC) or ^177^Lu-[1,4,7,10-tetraazacyclododecane-*N*,*N′*,*N″*,*N″′*-tetraacetic acid^0^-d-Phe^1^,Tyr^3^]octreotate (^177^Lu-DOTATATE) has recently been described [33–35]. Therefore, there might be a therapeutic approach to GBM using radiolabeled somatostatin analogs.

The aim of this study was to assess SSTR2A expression of both malignant glioma cells as well as macrophages in GBM as a potential target for imaging and therapy. Additionally, neuropathological findings were correlated to SSTR-PET imaging in three patients to evaluate potential feasibility of PET in diagnosis and therapy planning.

## Materials and Methods

### Samples and histological characterization of tumors

We retrospectively evaluated specimens resected from 15 patients (10 males, 5 females) with the initial diagnosis of glioblastoma multiforme (WHO grade IV) who underwent tumor resection at the Department of Neurosurgery of the University Hospital Würzburg between January 2012 and March 2013. Informed written consent was obtained from all patients.

All tumors were histologically assessed and graded on formaline fixed and paraffin embedded tissue sections by an experienced neuropathologist (CMM), according to the criteria of the World Health Organisation [36]. All specimens included the core of the tumors. In 14/15 cases (all samples except patient #1), the infiltration zone was examined for intra-tumoral heterogeneity of receptor expression. Paraffin sections (3 μm thick) were stained by applying immunohistochemical methodology. Glial origin of the tumor cells was confirmed by the positive reaction with the glial fibrillary acid protein (GFAP,1:200, Clone 6F2, Dako, Hamburg, Germany). Neurons and neuropile islands within tumor samples were identified by immunohistochemical staining with an antibody against synaptophysin (1:50, Clone SY38, Dako, Hamburg, Germany). To determine the proliferative activity of tumor cells, Ki-67 labeling index was calculated after immunostaining for MIB-1 (monoclonal, clone Ki-67, 1:50, Dako, Hamburg, Germany) by determining the number of positive nuclei among 100 tumor cells per high power field (HPF) (x400) in a total of 10 HPF per sample. An anti-CD68-antibody (1:200, Clone IS609, Dako, Hamburg, Germany) was used to identify intratumoral microglial cells and macrophages. Furthermore, to distinguish between local resident microglia and infiltrating macrophages, staining for the Leucocyte Common Antigen (CD45, 1:100, Clone 2B11+PD7/26, Dako, Hamburg, Germany) was performed. Sections were incubated overnight at 4°C with the primary antibody. Antibody-binding was visualized using a labeled streptavidin-biotin complex (LSAB, DCS, Hamburg, Germany) and the Envision System-horseradish peroxidase (HRP) (DAB, Dako, Hamburg, Germany). All immunostained sections were counterstained for 2 minutes with hematoxylin in order to identify tumoral or underlying parenchyma cells. A visual semiquantitative relative assessment of macrophages and malignant glioma cells was done. For this purpose, the fraction of labeled cells was graded using a scale from 0–20%, 20–50% and >50% in correspondence to the visual grading “few”, “moderate” and “numerous”, respectively, relative to the cell amount of the entire tumor sample.

### SSTR2A immunohistochemistry

Adjacent paraffin sections (3 μm thick) were used for immunohistochemistry with a polyclonal antibody against SSTR2A (1:100, Zytomed, Berlin, Germany) and with a monoclonal antibody against SSTR2A (1:200, Clone LS-C75923, LifeSpan BioScience, Eching, Germany). Samples from normal pancreas tissue were used as positive control, as recommended by the manufacturer. Dewaxed samples were pretreated with the antigen retrieval agent Target Retrieval Solution (TRS) pH 9.0 (Dako, Hamburg, Germany; Tris/EDTA) for 10 minutes in a high pressure cooker. Thereafter, they were washed in TBS pH 7.6 followed by peroxidase blocking with 2.5% H_2_O_2_ for 15 min. Serum blocking was performed for 20 min. using 10% normal goat serum (Invitrogen 50062Z, Darmstadt, Germany). For detection, link- and label-antibody from the SS Multilink HRP kit (DCS, LP000-UL, Hamburg, Germany) and the Romuline-AEC-Chromogen-kit (Zytomed, RAEC810L, Berlin, Germany) or the ultraView Universal DAB Detection Kit (Ventana Medical Systems, 760–500, Darmstadt, Germany) were used according to the manufacturer's instructions. All immunostained sections were counterstained for 2 minutes with hematoxylin. Sections without exposure to the primary antibodies served as negative controls as well as cross-sections exposed to human normal serum. Cross-sections derived from human normal pancreas selectively staining pancreatic islet cells were used as positive controls.

### Imaging

In 3 patients (#2, 3 and 15), the possible relationship of ^68^Ga-DOTATATE-uptake and the density of macrophages/microglia within the malignant glioma was investigated. For this purpose, routine magnet resonance (MR) imaging and SSTR-PET/CT with ^68^Ga-DOTATATE serving as tracer was performed. One patient (#15) also underwent amino acid based PET with O-(2-^18^F-fluoroethyl)-l-tyrosine (^18^F-FET). PET imaging was performed in the clinical routine as part of the pre-operative diagnostic work-up and the imaging test was offered to patients on the basis of compassionate use; written informed consent for the imaging procedures was obtained. Consequently, additional approval was waived by the local ethics committee (University of Würzburg).

### Non-invasive imaging of SSTR-2 expression and amino acid transport in patients with glioma

Synthesis of the SSTR-2 specific ligand ^68^Ga-DOTATATE was carried out on a computer-assisted synthesis module (Scintomics, Fürstenfeldbruck, Germany) using a modification of the method previously described by Breeman et al. [37].

Imaging of SSTR expression was performed on a state-of-the-art PET/CT device (Siemens Biograph mCT 64, Siemens, Knoxville, USA) consisting of a Lutetium oxyorthosilicate full-ring PET scanner and a 64-slice spiral CT. ^68^Ga-DOTATATE (121 ± 12 MBq) was injected intravenously. After 40–60 min, transmission data were acquired using spiral CT (80 mAs, 120 kV, a 512 × 512 matrix, 5 mm slice thickness, increment of 30 mm/s, rotation time of 0.5 s, and pitch index of 0.8). Consecutively, PET emission data were acquired in three-dimensional mode with a 200 × 200 matrix with 2 min emission time per bed position. After decay and scatter correction, PET data were reconstructed iteratively with attenuation correction using a dedicated software (HD-PET, Siemens Esoft, München, Germany).

^18^F-FET was synthesized in-house on a GE TRACERlab FX-FN synthesis module (GE Medical Systems, Uppsala, Sweden) in analogy to the method previously described by Bourdier et al. [38]. PET/CT was performed according to the guidelines for brain tumor imaging [39]. Static PET images were acquired 30 min after intravenous injection of 237 MBq ^18^F-FET. The scan lasted for 10 min. Data acquisition as well as reconstruction parameters were the same as for SSTR-PET/CT.

### Magnetic resonance imaging

All 3 patients underwent routine MR imaging (3TMagnetom Trio MR; Siemens, München, Germany) with a standard head coil (T1, T2, and FLAIR sequence). Axial T1-weighted images were obtained from the second cervical vertebral body to the vertex. Additionally, after intravenous administration of contrast agent gadoteric acid (DOTAREM; Guerbet, Villepinte, France), three-dimensional T1 weighted gradient echo sequences were performed for use in intraoperative neuronavigation.

### Image interpretation and data analysis

All imaging tests were reviewed independently and SSTR-PET findings were compared to MRI and/or ^18^F-FET-PET results. All studies were analyzed by two experienced nuclear medicine physicians (C.L., K.H.) who were aware of the clinical data.

^68^Ga-DOTATATE and ^18^F-FET uptake in the tissue were calculated as standardized uptake value (SUV) by dividing the radioactivity (kBq/mL) in the tissue by the radioactivity injected per gram of body weight.

Image analysis was performed as previously described [40]. In short, images were first inspected visually. Then the axial PET image displaying the maximum tumor uptake was selected. Tumor regions of interest (ROIs) were defined in 2 ways. Firstly, a standardized 10 mm circular region was placed over the area with the peak activity. This initial ROI was used to derive the maximum standardized uptake value (SUV_max_) and the mean standardized uptake value. A reference region in the normal brain was defined by drawing a ROI (diameter of 25 mm) involving the entire contralateral hemisphere at the level of the centrum semiovale to derive tumor-to-background ratios (TBR_mean_ and TBR_max_).

### Correlation of imaging results with histopathology

In three patients (#2, 3 and 15), ^68^Ga-DOTATATE-PET/CT and MR images were transferred to a neuronavigation system (Stealth Station S7, Medtronic Navigation, Louisville, USA) and combined in image fusion planning (StealthMerge Image Registration, Medtronic Navigation, Louisville, USA). During surgery of the gliomas, neuronavigated biopsy specimens were separately obtained from areas with visually different ^68^Ga-DOTATATE-uptake by an experienced neurosurgeon (M.L.) 3 samples out of areas with high, moderate and/or low ^68^Ga-DOTATATE uptake were acquired from each patient and checked for macrophage infiltration. A total of nine samples (3 per patient) were examined. After histological processing, the percentage of microglia and macrophages was visually assessed, calculated and graded using the following scale: 0–20%, 20–50% and >50% related to the total number of cells assessed for SSTR2A staining. For each patient, 3 different tumor areas with a diameter of 5 to 10 mm were examined and correlated with the ^68^Ga-DOTATATE uptake level (SUV_max_).

## Results

### Histopathological characterization of resected tumor samples

Specimens from 15 patients (10 males, 5 females) with the initial diagnosis of glioblastoma multiforme were resected and subsequently analyzed. At time of diagnosis, the mean age of the subjects was 62±12 y (range, 42–83 y). 6/15 tumors were located frontal and, 1/15 fronto-parietal. 2/15 patients presented with parietal glioma, 1/15 with temporo-parietal GBM. In each 2/15 patients the tumors were located temporal (n = 2) or occipital (n = 2), respectively; the remaining patient (1/15) suffered from temporo-occipital GBM. Symptoms varied according to malignant glioma location within the brain and included motor weakness, visual symptoms, language deficits, cognitive and personality changes as well as headaches and seizures. None of the patients had undergone any previous treatment. Complete surgical resection could be performed in 5/15 patients, in the remainder (10/15) only sub-total or partial resection was achieved according to pathological analysis and/or post-operative imaging.

11/15 patients died from GBM during follow-up (mean overall survival, 11±6 months), the remaining 4 subjects are still alive.

13/15 tumor samples (87%) demonstrated a tumor cell burden >50%. In the remaining two samples, malignant glioma cells comprised 20–50% of total cells. In all specimens, considerable numbers of tumor associated macrophages could be detected: In 4/15 cases (27%), less than 20% of cells stained for CD68; in 10/15 samples (67%) infiltration was > 20% but < 50% and in the remaining 1/15 samples (7%), more than 50% of the cells represented tumor associated macrophages. To identify infiltrating monocytes/macrophages and lymphocytes (CD45^high^) and to distinguish these from residential microglia cells (CD45^low^), tissue sections were analyzed for CD45 expression. In agreement with current literature, resident/ramified microglia had a fainter CD45 immunostaining compared to recruited leukocytes which impressed as round-globoid cells with strong immunoreactivity. However, in all 15 glioblastoma-specimens, few CD45^high^ cells were found, whereas numerous CD45^low^ cells were present, indicating a low number of tumor-infiltrating macrophages (S1 Fig.).

Cell proliferation, as assessed by MIB-1 (clone Ki-67), was lower than 50% in all samples. Details are given in Table 1.

**Table 1:** Distribution of macrophage infiltration, tumor cell burden and proliferation (visual assessment) in GBM samples.

Patient	Age (years)	Sex	Macrophage infiltration (%)	Tumor cell (%)	Ki 67 (%)
**1**	72	F	20–50	>50	25–30
**2**	83	F	Table 2	Table 2	20–30
**3**	69	M	Table 2	Table 2	20–30
**4**	51	F	20–50	>50	15–25
**5**	72	M	20–50	>50	20
**6**	48	M	<20	>50	30
**7**	56	M	20–50	>50	10–20
**8**	70	M	20–50	>50	25
**9**	77	F	<20	>50	40–50
**10**	42	M	>50	20–50	15
**11**	62	M	<20	>50	15–30
**12**	60	M	20–50	>50	25
**13**	72	M	20–50	>50	15–20
**14**	51	M	20–50	>50	15–20
**15**	51	F	Table 2	Table 2	10–15

### Expression of somatostatin receptor 2A (SSTR2A) in resected tumor samples

**Fig 1:**
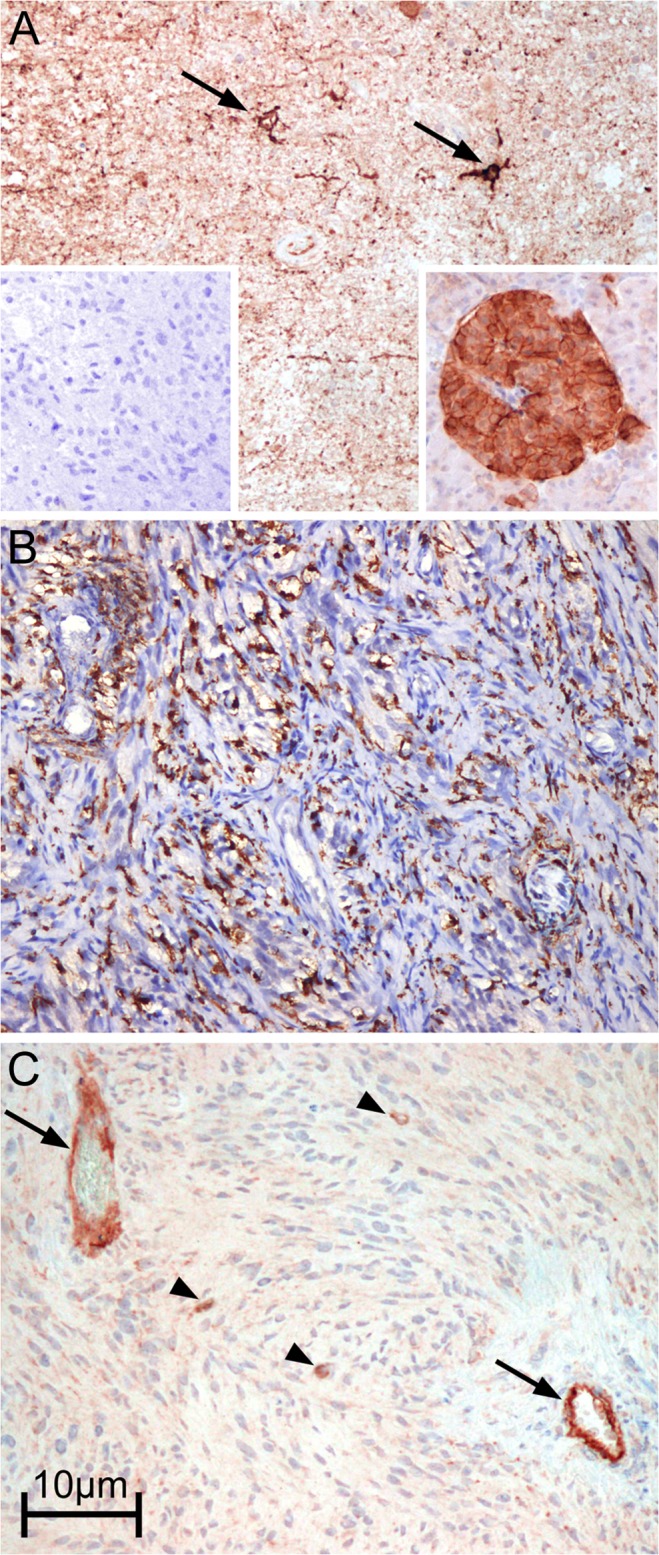
Immunohistochemical staining of SSTR2A (sample of patient #15). (A) non-tumoral cortex with strong reaction in neurons (arrows) and neuropile (magnification: 100x). Left inset: Negative control. Right inset: positive control (normal pancreas) with strong reaction in an islet of Langerhans. (B) CD68 staining depicting high microglia/macrophage infiltration area and (C) SSTR2A immunostaining of an adjacent area showing strong reaction in endothelium (arrows) and in single glioma cells (arrowheads) (magnification: 100x).

Only faint SSTR2A immunostaining of single macrophages was detected independently of the antibody used; therefore, no correlation to the relatively higher number of macrophages could be demonstrated (Fig. 1). No clear differences between tumor-infiltrating macrophages and resident microglial cells could be established. In addition, only single tumor cells showed a similarly weak staining for SSTR2A. The staining was confined to the membrane and cytoplasma whereas the nucleus was spared. Quantification was not feasible due to the very low number of immunopositive cells (lower than 10%). In 14/15 cases (all except sample of patient #1), the tissue sections included both the central core as well as invasive margins of the tumor (infiltration zone). No difference regarding SSTR2A expression between both areas was observed with a very low number of SSTR-positive cells in all areas. In fact, a strong positive reaction was seen only in adjacent cortical areas (in neurons and neuropile) and in entrapped neurons in some tumor areas (S2 Fig.).

In contrast, in all samples examined a strong SSTR2A staining of intratumoral vascular endothelial cells, adjacent neuropile and neuronal cells could be observed. Non-tumoral astrocytes and oligodendrocytes, on the other hand, did not express SSTR2A. In some cases, a fine granular staining was observed also in the protoplasmic background of the tumor tissue.

A representative example of immunostaining in one resected tumor sample is given in S3 Fig. (monoclonal and polyclonal anti-SSTR2-antibody).

### SSTR-PET imaging of glioblastoma multiforme

**Fig 2:**
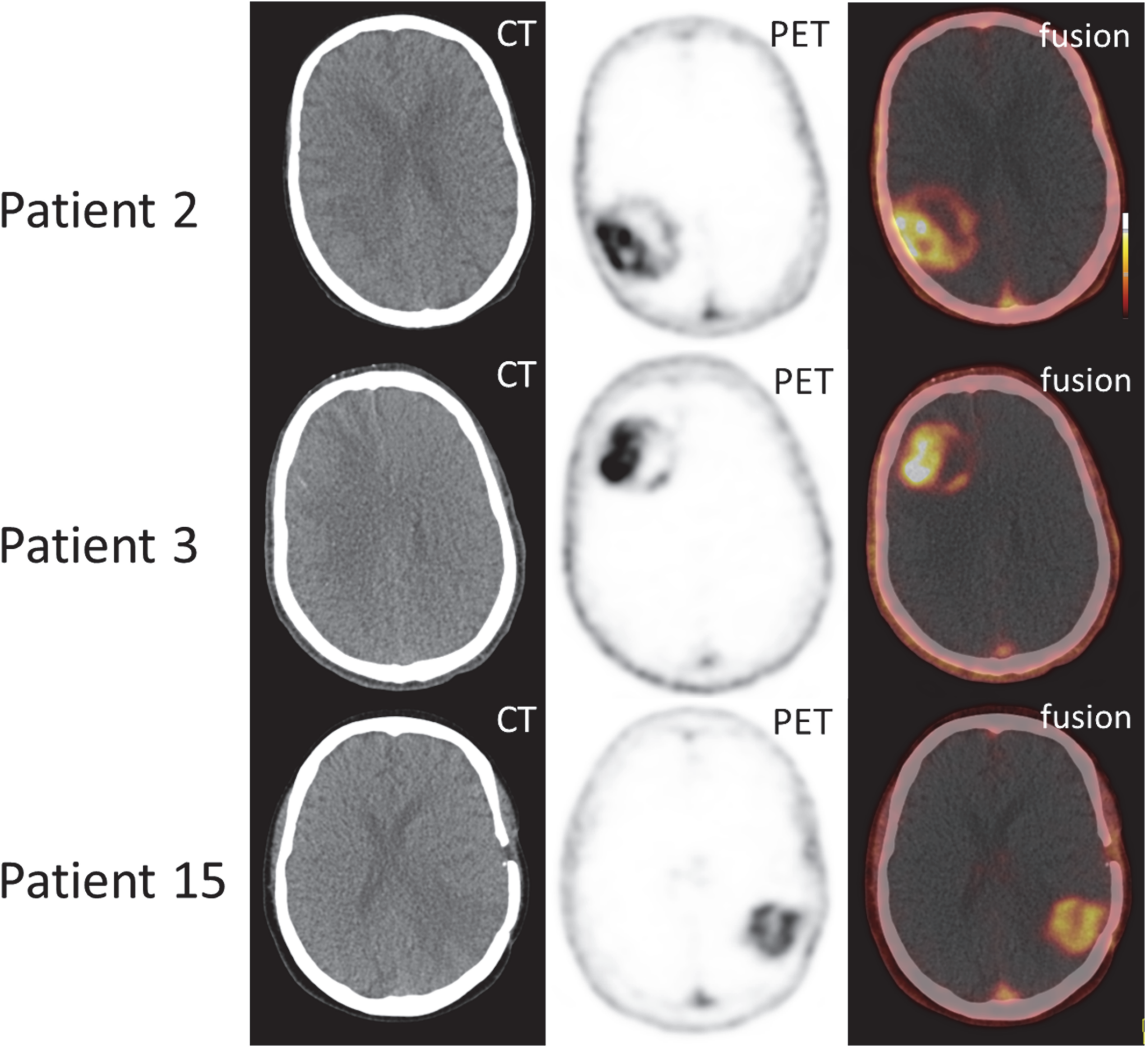
Tracer distribution of ^68^Ga-DOTATATE in GBM. Three examples of increased ^68^Ga-DOTATATE uptake in Glioblastoma multiforme. Markedly, partly inhomogeneous tracer accumulation can be depicted in each patient. Shown are axial views of CT, SSTR-PET as well as fused PET/CT images.

**Fig 3:**
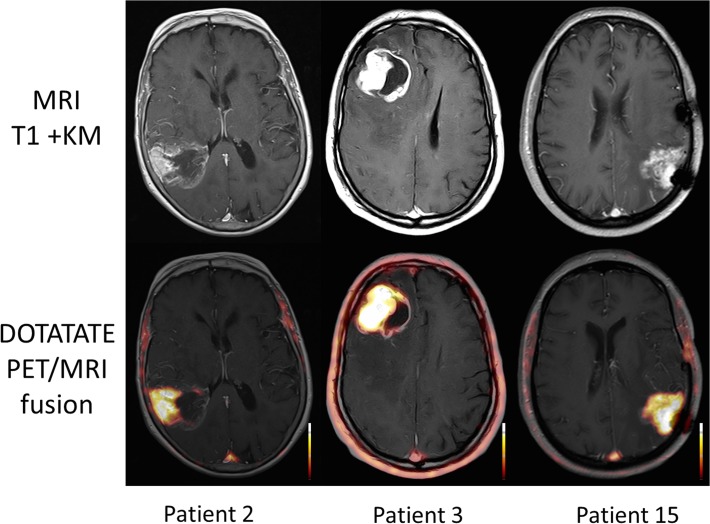
Comparison of ^68^Ga-DOTATATE uptake and MR contrast agent enhancement. In all 3 patients, there is a marked overlap between ^68^Ga-DOTATATE uptake and MR contrast agent enhancement. Radiotracer accumulation is most pronounced in areas with intense contrast enhancement. Shown are axial views of contrast-enhanced T1-weighted MR as well as fused MR/PET images.

**Fig 4:**
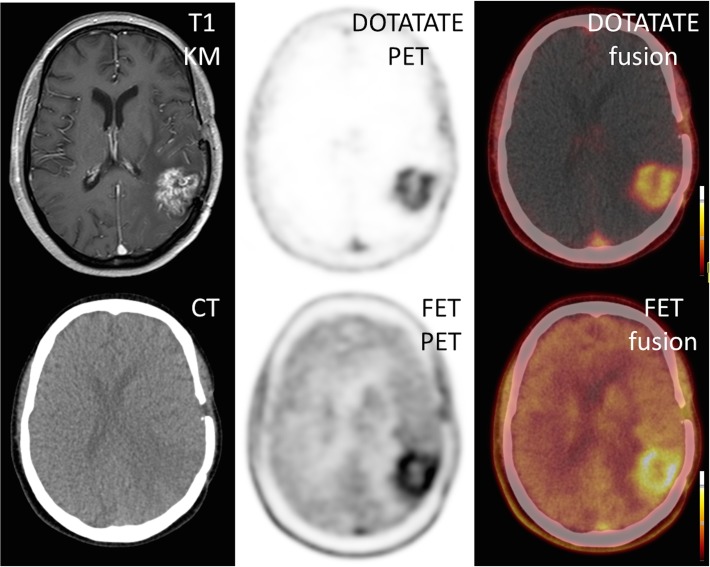
Comparison of ^68^Ga-DOTATATE and ^18^F-FET in GBM. A single patient (#15) underwent both SSTR- as well as amino acid-based PET. Both modalities show highly comparable tracer uptake.

All 3 patients who underwent SSTR-PET/CT showed areas of markedly increased ^68^Ga-DOTATATE uptake (Fig. 2). Median SUV_mean_ was 3.9 (range 3.5–4.5), median SUV_max_ 5.1 (range 4.5–5.8), median TBR_mean_ 193 (range 175–225) and median TBR_max_ was 130 (range 116–175). Those excellent tumor-to-background contrasts are due to extremely low background activity, ranging from 0.02 to 0.05. Interestingly, ^68^Ga-DOTATATE uptake was highest in areas with distinct contrast enhancement on MR imaging (Fig. 3). On visual inspection, the pattern of ^68^Ga-DOTATATE uptake matched ^18^F-FET accumulation regarding size and shape (Fig. 4). However, ^68^Ga-DOTATATE allowed better tumor delineation than ^18^F-FET: In the one patient (# 15) undergoing both scans, TBR_mean_ und TBR_max_ were 180 and 125 for ^68^Ga-DOTATATE and 3.9 and 3.6 for ^18^F-FET, respectively.

### Comparison of ^68^Ga-DOTATATE uptake and histopathological findings

The percentage of tumor cells ranged from <20–>50% with >50% in 6/9 of samples (3 samples per patient #2, 3, 15). In one surgery specimen (1/9; #3.3), numbers ranged from 20–50%, in the remaining 2 cases (2/9; #3.1, 15.3), malignant glioma cells made up less than 20% of the sample.

Macrophage numbers were also variable, ranging from <20% to >50%. In 4/9 specimens, tumor-associated macrophages comprised less than 20% of cells. In 4/9 samples 20–50%, and in the remaining specimen more than 50%, respectively. However, the number of SSTR-positive cells did not exceed 10% in any sample independent of tracer uptake. Therefore, no correlation between macrophage or malignant glioma cell numbers and tracer accumulation was observed (Table 2).

**Table 2:** Correlation between histological macrophage infiltration (visual assessment) and ^68^Ga-DOTATATE uptake in three different samples of GBM.

**Case 2 Sample no**.	**Macrophage assessment (% of entire sample)**	**Tumor cells (% of entire sample)**	**SSTR2 positive cells (%)**	^**68**^ **Ga-DOTATATE uptake level**
**1**	20–50	>50	<10	high
**2**	<20	>50	<10	low
**3**	20–50	>50	<10	none
**Case 3 Sample no**.	**Macrophage assessment (% of entire sample)**	**Tumor cells (% of entire sample)**	**SSTR2 positive cells (%)**	^**68**^ **Ga-DOTATATE uptake level**
**1**	<20	<20	<10	moderate
**2**	20–50	>50	<10	high
**3**	<20	20–50	<10	low
**Case 15 Sample no**.	**Macrophage assessment (% of entire sample)**	**Tumor cells (% of entire sample)**	**SSTR2 positive cells (%)**	^**68**^ **Ga-DOTATATE uptake level**
**1**	>50	>50	<10	high
**2**	20–50	>50	<10	high
**3**	<20	<20	<10	moderate

## Discussion

Tumor-associated macrophages (TAM) are generally known to promote the invasion, growth, and angiogenesis of various tumors. This is the first report to assess the expression of somatostatin receptor 2A in TAMs in glioblastoma multiforme. In our cohort, immunohistochemistry managed to depict CD68-positive infiltrates comprising up to 20–50% of the individual tumor samples. This finding is in line with the literature reporting that TAMs can comprise up to 40% of all cells in GBM [7, 8]. Therefore, we hypothesized some role for SSTR-PET/CT in the depiction of macrophage infiltration and, thus, individual prognosis. Additionally, since local peptide receptor-mediated radiotherapy in GBM has been described [31–33], feasibility of a “theranostic” approach using SSTR-PET/CT for evaluation of receptor expression and consecutive systemic peptide radio receptor therapy was to be assessed. However, robust SSTR2A staining was observed only in single macrophages. This finding is not in line with previous reports that found a high expression of SSTR2A on the surface of peripheral activated macrophages *in vitro* [26]. However, as the other groups did not investigate glioma tissues, final conclusions cannot be drawn at this time.

Several authors have reported a robust somatostatin receptor expression on the surface of malignant glioma cells [28–30]. However, in our series of GBM samples with considerable tumor cell burden (>50% in all but two samples), we could not detect any relevant receptor expression on the GBM tumor cell surface. On the contrary, SSTR2A showed a higher expression in the normal brain parenchyma including neurons and vessels. This finding was consistent in both the tumor core as well as the infiltration zone.

Interestingly, in the three patients who underwent SSTR-PET/CT prior to brain surgery, ^68^Ga-DOTATATE uptake within tumor tissue could be observed in all patients. However, intensity of radiotracer accumulation did not correlate with macrophage density in histology. On the contrary, a better correlation of ^68^Ga-DOTATATE uptake could be observed with contrast enhancement in magnetic resonance imaging, thereby questioning tracer specificity. In the light of these findings, reports on successful treatment of GBM with ^90^Y-DOTATATE on the basis of a positive pre-therapeutic PET scan cannot be interpreted as examples of targeted therapies [33]. Although some therapeutic effects can be achieved due to high radiation doses delivered directly in the surgical cavity, the notion that those effects are achieved by specific binding of radiopharmaceutical to TAMs and/or malignant glioma cells is highly questionable. On the contrary, somatostatin receptor-targeted tracer uptake in GBM seems to be highly unspecific and caused by the disruption of the blood-brain barrier.

This study has several limitations. A rather small sample size was included. Since the tumors were not examined *in toto*, histology is prone to sampling bias. Macrophage labelling with CD68 specific antibodies does not necessarily allow to discriminate macrophages from tumor cells since both cell types may present with positive staining patterns [41,42]. However, SSTR expression was not sufficient in all samples so that the assignment of receptor expression to the tumor cell compartment or to cells of the immune system seems less relevant in our series. PET imaging was available in only three patients. Since we did not perform further experiments regarding macrophage differentiation, the reason for the obvious loss of the surface receptor can only be hypothesized. It is known that TAM may differentiate into two profiles, M1 and M2 [43, 44]. The classical activation is caused by stimuli like interferon-γ or tumor-necrosis-factor-α and results in a pro-inflammatory activation state called M1. The alternative pathway is induced by interleukin-4 or interleukin-13 and leads to a rather immunosuppressive state. This M2 polarization has been found to correlate with glioma malignancy and to represent the predominant macrophage population in GBM. This condition is linked to increased tumor invasiveness, higher glioma proliferation rates and poorer prognosis [45, 46]. Potentially, M2 differentiation of macrophages within the tumor-microenvironment of GBM leads to a decreased SSTR expression. An optimal therapeutic scenario would include a high receptor expression by M2 and a low expression by M1 macrophages. However, we could not observe any relevant SSTR2A expression in all our samples (including tumor core as well as margins). As we focused on the potential of the receptor as a target for radiopeptide therapy, the question of macrophage differentiation is beyond the scope of the present study. To the best of our knowledge, there has not been any systematic assessment of this question so far.

On the other hand, it might be possible that only tumor-infiltrating macrophages express SSTR2A, whereas the resident microglia does not. In our series, we could identify few infiltrating cells so that a final conclusion concerning their receptor expression level cannot be drawn yet. However, since numbers of glioma-infiltrating monocytes/macrophages were low, they do not represent a good target for SSTR-based therapeutic interventions.

Although first results regarding local instillation of radiolabeled somatostatin analogs have yielded encouraging results, our findings question a role for peptide receptor-based therapy as a novel treatment approach in GBM. Furthermore, molecular imaging using SSTR-PET/CT seems not to be perfectly suited to elucidate the amount of TAMs. Even the possible application of ^68^Ga-DOTATATE to delineate tumor tissue (given its very similar distribution in comparison to ^18^F-FET) in settings in which a cyclotron is not available is rendered doubtful given the unspecific tracer distribution.

In conclusion, somatostatin receptor-directed imaging or therapy may represent a less favourable approach in glioblastoma multiforme. Larger trails are needed to further assess its use in the diagnostic or therapeutic management of GBM.

## Supporting Information

S1 Fig Immunohistochemical staining of CD45 (sample of patient #5) to distinguish between tumor-infiltrating monocytes/macrophages (CD45^high^) and resident microglia (CD45^low^). (A) Few CD45^high^ cells are discernable, indicating a low number of tumor-infiltrating macrophages (magnification: 100x). (B) Weak staining (CD45^low^) in tumor associated microglia. These cells are well recognizable by their spindle-shaped (black arrows) or more ramified cytoplasm (red arrow) (magnification: 400x). (C) Strong staining (CD45^high^) in infiltrating macrophages. These cells possess a rounded-globoid cytoplasm (magnification: 400x).
(TIF)

S2 Fig Immunohistochemical staining of SSTR2A (sample of patient #8) including (A) the core of the tumor and (B) the infiltration zone (magnification: 100x). No significant expression of SSTR2A by tumor cells or macrophages could be detected. Instead, SSTR2A-positivity could be detected in (A) normal vessel walls and (B) in neurons and neuropile (arrows). Panel (C) gives an example of a strongly stained neuron (magnification: 200x).
(TIF)

S3 Fig A representative example of immunostaining in one resected tumor sample with (A) a monoclonal and (B) a polyclonal anti-SSTR2A-antibody (sample of patient #3). With both antibodies, only few SSTR2A-positive cells can be detected (magnification: 100x).
(TIF)
