# Supplementary figures and images for: Tumor-Associated Macrophages in Glioblastoma Multiforme—A Suitable Target for Somatostatin Receptor-Based Imaging and Therapy?
Source: PLoS One. 2015 Mar 25;10(3):e0122269. doi: 10.1371/journal.pone.0122269 (PMC4373835; doi:10.1371/journal.pone.0122269)

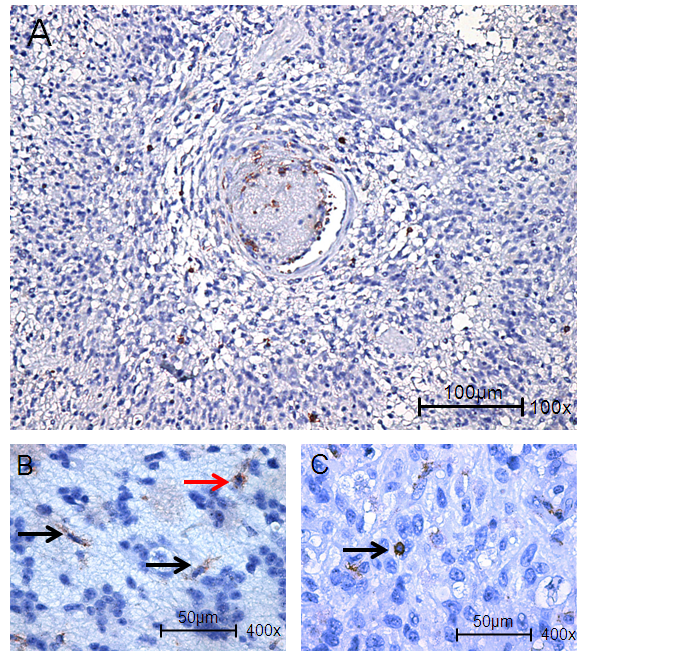

Supplement: S1 Fig — (A) Few CD45high cells are discernable, indicating a low number of tumor-infiltrating macrophages (magnification: 100x). (B) Weak staining (CD45low) in tumor associated microglia. These cells are well recognizable by their spindle-shaped (black arrows) or more ramified cytoplasm (red arrow) (magnification: 400x). (C) Strong staining (CD45high) in infiltrating macrophages. These cells possess a rounded-globoid cytoplasm (magnification: 400x). (TIF) [file pone.0122269.s001.TIF]

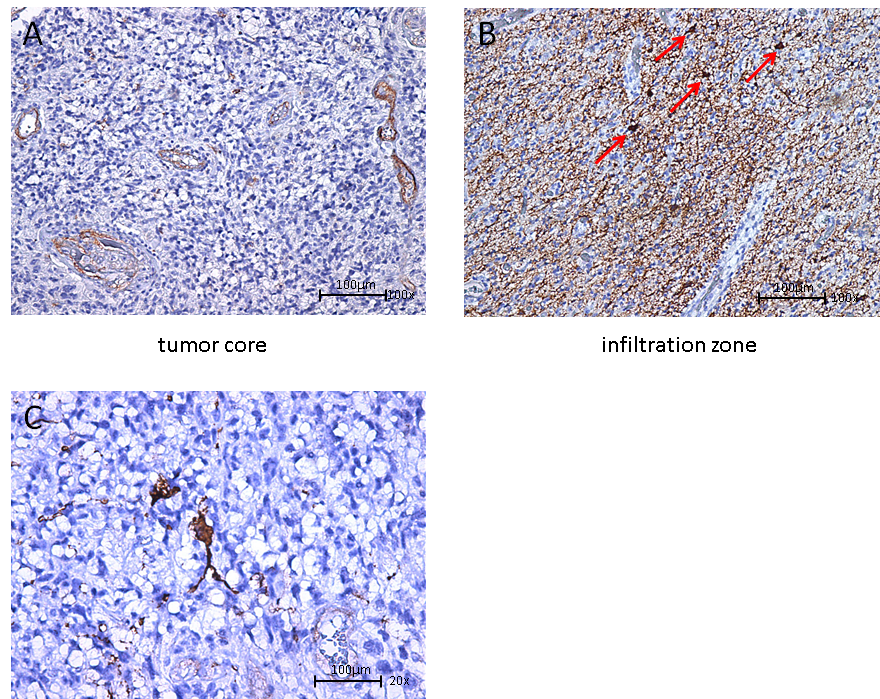

Supplement: S2 Fig — No significant expression of SSTR2A by tumor cells or macrophages could be detected. Instead, SSTR2A-positivity could be detected in (A) normal vessel walls and (B) in neurons and neuropile (arrows). Panel (C) gives an example of a strongly stained neuron (magnification: 200x). (TIF) [file pone.0122269.s002.TIF]

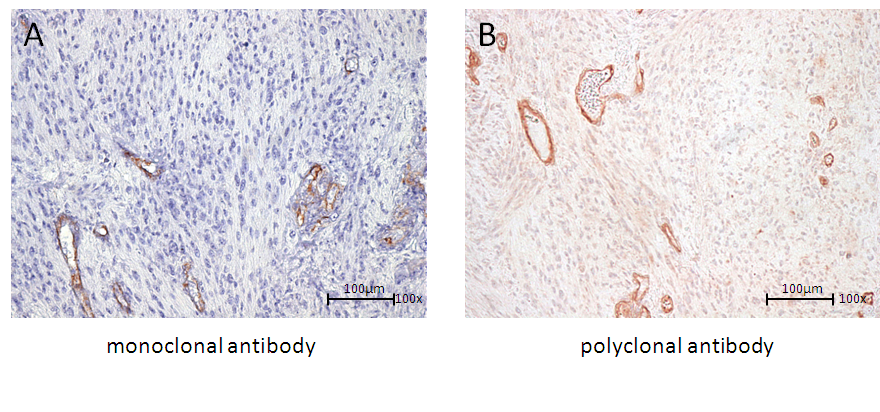

Supplement: S3 Fig — With both antibodies, only few SSTR2A-positive cells can be detected (magnification: 100x). (TIF) [file pone.0122269.s003.TIF]
